# Chronic Urotensin-II Administration Improves Whole-Body Glucose Tolerance in High-Fat Diet-Fed Mice

**DOI:** 10.3389/fendo.2019.00453

**Published:** 2019-07-12

**Authors:** Xi Chen, Lin Yin, Wei-hua Jia, Nuo-qi Wang, Chun-yang Xu, Bi-yu Hou, Na Li, Li Zhang, Gui-fen Qiang, Xiu-ying Yang, Guan-hua Du

**Affiliations:** ^1^State Key Laboratory of Bioactive Substance and Function of Natural Medicines and Beijing Key Laboratory of Drug Target and Screening Research, Institute of Materia Medica of Peking Union Medical College, Beijing, China; ^2^College of Pharmacy, Harbin University of Commerce, Haerbin, China

**Keywords:** urotensin-II, high-fat diet, glucose tolerance, skeletal muscle, adipose tissue, mitochondrion

## Abstract

Urotensin-II (U-II) is an endogenous peptide agonist of a G protein-coupled receptor—urotensin receptor. There are many conflicting findings about the effects of U-II on blood glucose. This study aims to explore the effects of U-II on glucose metabolism in high-fat diet-fed mice. Male C57BL/6J mice were fed a 45% high-fat diet or chow diet and were administered U-II intraperitoneally for *in vivo* study. Skeletal muscle C2C12 cells were used to determine the effects of U-II on glucose and fatty acid metabolism as well as mitochondrial respiratory function. In this study, we found that chronic U-II administration (more than 7 days) ameliorated glucose tolerance in high-fat diet-fed mice. In addition, chronic U-II administration reduced the weight gain and the adipose tissue weight, including visceral, subcutaneous, and brown adipose tissue, without a significant change in blood lipid levels. These were accompanied by the increased mRNA expression of the mitochondrial thermogenesis gene *Ucp3* in skeletal muscle. Furthermore, *in vitro* treatment with U-II directly enhanced glucose and free fatty acid consumption in C2C12 cells with increased aerobic respiration. Taken together, chronic U-II stimulation leads to improvement on glucose tolerance in high-fat diet-fed mice and this effect maybe closely related to the reduction in adipose tissue weights and enhancement on energy substrate utilization in skeletal muscle.

## Introduction

Urotensin-II (U-II) was first isolated from the neurosecretory system of the goby fish (*Gillichthys mirabilis*) (1) and later cloned from humans (2). U-II is a potent endogenous urotensin receptor (UT receptor) peptide agonist (3), which is a G protein-coupled receptor with Gαq signal transduction. U-II isopeptides have a cyclical structure and are 11–15 amino acids in length. The human U-II (hU-II) having been identified in the spinal cord is 11 amino acids long. The UT receptor, also known as GPR14, is involved in the regulation of vasoconstriction (3, 4). In mammals, the mRNA transcripts for the U-II precursor (*UTS2*) and UT receptor gene (*UTS2R*) are widely expressed in the central nervous system and in peripheral tissues, including the brain, kidney, liver, lung, pancreas, skeletal muscle, and other tissues and vascular and cardiac cells (5, 6). Consistent with the wide distribution of U-II and UT receptors, the urotensinergic system has been linked with numerous pathological states, including atherosclerosis, heart failure, hypertension, renal disease, and diabetes (7).

Several studies have shown that the urotensinergic system is closely related to glucose and fat metabolism (8–13). However, the effect remains controversial. Increased plasma U-II levels in patients with diabetes mellitus (8) and elevated expression of *Uts2* and its receptor in the skeletal muscle of diabetic mice (10) were reported. In *Uts2* knockout mice, there was a significant reduction in weight gain, visceral fat, blood pressure, and circulating plasma lipids and an improvement in glucose tolerance compared with those in wild-type mice (11). Blocking the UT receptor pathway ameliorated metabolic syndrome, and the weight gain of ob/ob mice was significantly greater than that of wild-type mice (12). U-II could reduce the glucose-evoked insulin release (14). Studies have reported that selective UT receptor antagonists could improve glucose tolerance (12, 15). However, there are also many conflicting reports. Sheridan et al. (16) reported that U-II tended to induce hypoglycemia in coho salmon. In *Uts2r* knockout mice, serum glucose was similar to that in wild-type mice (17). Shiraishi et al. (18) also reported that U-II administrations with a dosage of 500 pmol/kg body weight per hour for a total of 4 weeks had no significant effect on glucose or insulin levels. Mice which are homozygous for the *Uts2r* gene deletion have elevated serum triglyceride and cholesterol levels compared to wild-type controls (17, 19). When crossed with mice carrying a knockout mutation of the *Apoe* gene, the resulting double mutant mice exhibited more severe atherosclerosis, hyperlipidemia, and hyperinsulinemia compared to *Apoe* knockout mice (17, 19).

Given the contradictory results, it is necessary to conduct research on the effects of U-II on glucose and lipid metabolism. In this study, we used normal and high-fat diet (HFD)-fed mice to evaluate the effects of U-II, focusing on the glucose tolerance investigation.

## Materials and Methods

### Reagents

U-II (HPLC, >98%) was provided as a lyophilized powder by GL Biochem Ltd., (Shanghai, China). The blood-glucose test used an Accu-Chek Active meter from Roche (Basel, Switzerland). Plasma triglyceride (TG), cholesterol (CHO), low-density lipoprotein (LDL), high-density lipoprotein (HDL), pyruvate, and fructosamine levels were detected with a commercially available enzyme kit (BioSino Bio-technology & Science Inc., Beijing, China). Lactic acid (LA) level kits were obtained from Nanjing Jiancheng Bioengineering Institute (Nanjing, China). An insulin ELISA kit was purchased from Mercodia Inc., (Uppsala, Sweden). High-fat diets were purchased from HFK Bioscience Co., Ltd., (Beijing, China). The quantification of DPP4 and glucagon in plasma was achieved by a high-performance Luminex^®^ assay from R&D Systems (Minneapolis, MN, USA). Cell viability was detected by CellTiter-Glo^®^ luminescent cell viability assay purchased from Promega Corporation (Madison, WI, USA). The glucose uptake of cells was detected using 2-(N-(7-nitrobenz-2-oxa-1,3-diazol-4-yl)amino)-2-deoxyglucose (2-NBDG) purchased from Thermo Fisher Scientific (Waltham, CA, USA). Oligomycin was purchased from Abcam (Cambridge, UK). Carbonyl cyanide-p-trifluoromethoxyphenylhydrazone (FCCP), antimycin A, and rotenone were obtained from Sigma (St. Louis, MO). MitoTracker Green FM was purchased from Thermo Fisher Scientific (Waltham, CA, USA). TRIzol isolation reagent was obtained from Invitrogen (Carlsbad, CA, USA). Direct-zol RNA kits were obtained from ZYMO research (Irvine, CA, USA). SuperScript III reverse transcriptase was obtained from Invitrogen (Carlsbad, CA, USA). SsoFast™ EvaGreen^®^ supermix was obtained from Bio-Rad (Hercules, CA, USA).

### Animal Care and Use

Male C57BL/6J mice (20–22 g) were obtained from the Institute of Laboratory Animal Science, Chinese Academy of Medical Sciences (Beijing, China). The animals were kept under a 12-h light/dark cycle at a temperature of 22 ± 3°C and a humidity of 55 ± 5%. Mice were given free access to food and water for 7 days before the experiment. All animal procedures were approved by the animal care and use committee of the Institute of Materia Medica, Chinese Academy of Medical Sciences, and were carried out strictly in accordance with research guidelines for the care and use of laboratory animals.

### U-II Treatments in Mice

Treatments of chow diet-fed mice: Mice were treated with two administration strategies. One was single administration, and the other was a chronic administration strategy. Male C57BL/6J mice (20–22 g) were randomly subdivided into control and indicated dosage U-II groups. All mice were given drugs via intraperitoneal injection (IP). Age-matched normal mice were received an equal volume of normal saline (NS). In single U-II administration experiment, U-II (0.1–100 nmol/kg) were given via IP (*N* = 7 in each group). After U-II administration for 15 min, the blood-glucose test, IPGTT, or ITT was performed, respectively. In chronic U-II administration experiment, U-II (20 nmol/kg) was given for 7 days via IP with normal saline administration as control (*N* = 15 in each group). U-II (20 nmol/kg) was given once per day by IP in morning. After 7 days, five mice from each group were taken for IPGTT and ITT separately. And the remaining five mice in each group were used for tissue collection.

Treatments of HFD-fed mice: Male C57BL/6J mice were randomly divided into a normal control group (7 mice) and a HFD group (40 mice). The mice in the HFD group were fed a 45% high-fat diet, and the mice in the normal group were fed a chow diet. Twelve weeks later, the mice in the HFD group with obesity (the body weight higher than 20% of the mean value of the body weight of the control mice) were chosen and randomly divided further into an HFD control group (7 mice) and a U-II treatment group (7 mice). U-II group mice were administered U-II intraperitoneally for 3 weeks. The mice in the normal control and HFD control groups were given NS. Blood samples were taken at the indicated times for tests from the tails of non-anesthetized mice.

### Cell Culture

C2C12 myoblasts (murine cell line) were purchased from ATCC (USA) and were cultured in high-glucose (25.5 mmol/L) DMEM with 5% FBS, 15% calf serum, 100 I.U./mL penicillin and 100 μg/mL streptomycin (Invitrogen, USA). The differentiation of myoblasts into myotubes was performed by incubating confluent myoblasts with differentiating media (2% horse serum, 100 I.U./mL penicillin, and 100 μg/mL streptomycin) for 4 days. Successful differentiation of the C2C12 cells was confirmed by morphological changes as previously reported (20). The culture protocol of C2C12 myoblasts was strictly enforced to avoid cell confluence.

### Glucose Uptake Detection

The method was performed according to the literature (21) with few modifications. In brief, cells were seeded in a 96-well, black, clear-bottom culture plate. Cells were maintained in serum-free media for 4–24 h before the addition of the testing agent. The 2-NBDG (Thermo, Waltham, CA, USA) stock solution was diluted to 100 μM with glucose-free, serum-free media. Culture media were discarded from the plates that were incubated, and the cells were washed twice with glucose-free media. A 2-NBDG solution was added with a concentration of 100 μL/well. Cells were incubated in the plate for 30 min in a 5% CO_2_ incubator at 37°C and then washed three times with 200 μL/well of ice-cold Hanks buffer. The fluorescence was measured using a microplate reader (Corning, NY, USA) (λex = 460–490 nm, λem = 530–550 nm).

### Quantitative Real-Time PCR

Total RNA was isolated using TRIzol isolation reagent (Invitrogen, USA) and then further purified with Direct-zol RNA kits (ZYMO Research, USA). First-strand cDNA was synthesized using 1.5 μg of total RNA with a reverse transcription reaction mix that contained SuperScript III reverse transcriptase (Invitrogen, USA) and Oligo-dT17 as primers. The expression of genes was detected using SsoFast™ EvaGreen^®^ supermix (Bio-Rad, USA) on a CFX-96 real-time PCR System (Bio-Rad, USA) with gene-specific primer pairs (Table 1). The results were quantified after normalization with TBP (22).

**Table 1:** Primers used for real-time PCR.

**Pairs**	**Genes**	**Primer sequence**
1	Mouse *Pgc1α* 5′	ATACCGCAAAGAGCACGAGAA
	Mouse *Pgc1α* 3′	CTCAAGAGCAGCGAAAGCGTCACA
2	Mouse *Tfam* 5′	CAGGAGGCAAAGGATGATTC
	Mouse *Tfam* 3′	ATGTCTCCGGATCGTTTCAC
4	Mouse *Ucp1* 5′	CCTGCCTCTCTCGGAAACAA
	Mouse *Ucp1* 3′	TGTAGGCTGCCCAATGAACA
5	Mouse *Ucp2* 5′	ATGGTTGGTTTCAAGGCCACA
	Mouse *Ucp2* 3′	CGGTATCCAGAGGGAAAGTGAT
6	Mouse *Ucp3* 5′	GCCTGTCATCGTCATCATCTAC
	Mouse *Ucp3* 3′	GCATGGCTTTACCACTACAAAC
7	Mouse *Ppara* 5′	CCTTGGTGCCATCCTCTCAG
	Mouse *Ppara* 3′	TGCCTGGAACCAATCAGCTC
8	Mouse *Tbp* 5′	ACCCTTCACCAATGACTCCTATG
	Mouse *Tbp* 3′	ATGATGACTGCAGCAAATCGC

### Mitochondrial Content

The method was performed per the manufacturer's instructions. In brief, C2C12 myoblasts were seeded in a black-wall, clear-bottom, 96-well cell culture plate with growth media. The next day, cells in 90–100% confluency were placed into differentiating media for 4 days. Afterwards, the differentiated myotubes were treated with the indicated drugs for 24 h. The media were removed, and pre-warmed (37°C) staining solution containing the MitoTracker^®^ probe (Thermo, Waltham, CA, USA) at a final concentration of 200 nmol/L was added for 30 min under differentiating-medium conditions. The staining solution was replaced with fresh pre-warmed media after staining was complete, and the result was detected with a fluorescence microplate reader (490 nm/516 nm).

### Mitochondrial Respiratory Detection in Cells

Mitochondrial respiration in C2C12 cells was determined using a Seahorse XFe96 Extracellular Flux analyser using the XF Mito stress test kit as previously described (23) (Agilent, USA). The concentrations of oligomycin, carbonyl cyanide-p-trifluoromethoxyphenylhydrazone (FCCP), antimycin A and rotenone used were 100, 100, 100, and 50 μmol/L, respectively. The oxygen consumption rate (OCR) and extracellular acidification rate (ECAR) were recorded, and the cellular respiration and ATP production were calculated as described by the manufacturer.

### IPGTT Detection

The glucose tolerance test (GTT) in mice was assessed by intraperitoneal GTT (IPGTT). In brief, after being fasted for 16 h, the blood-glucose levels were determined via blood collection from the tail vein as the 0-min blood-glucose level, and glucose (2.0 g/kg b.w.) was then administered by intraperitoneal injection to all mice. Blood sugar samples were collected at 15, 30, 60, and 120 min after glucose administration. Blood glucose was detected by the hexokinase method.

### ITT Detection

The insulin tolerance test (ITT) was tested by intraperitoneal injection of insulin. Mice were fasted for 4 h. Each mouse was weighed to calculate the necessary insulin needed for a dose of 0.75 I.U./kg body weight. The baseline blood-glucose level of each mouse was measured to obtain a reference value for 0 min. Mice were intraperitoneally injected at 0.05 mL/10 g body weight with a 0.15 I.U./mL insulin solution. Blood-glucose levels were determined by glucometer readings from the tail vein. Blood-glucose levels were measured at 15, 30, 60, and 120 min after insulin injection.

### Statistical Analysis

Results are expressed as the means ± S.E.M. and were considered significant at *P* ≤ 0.05. Statistical analysis was performed by unpaired two-tailed Student's test or analysis of variance, as appropriate (GraphPad Software, USA).

## Results

### U-II Single Administration Increased Blood Glucose and Caused Insulin Resistance in Chow Diet-Fed C57BL/6J Mice

**Figure 1:**
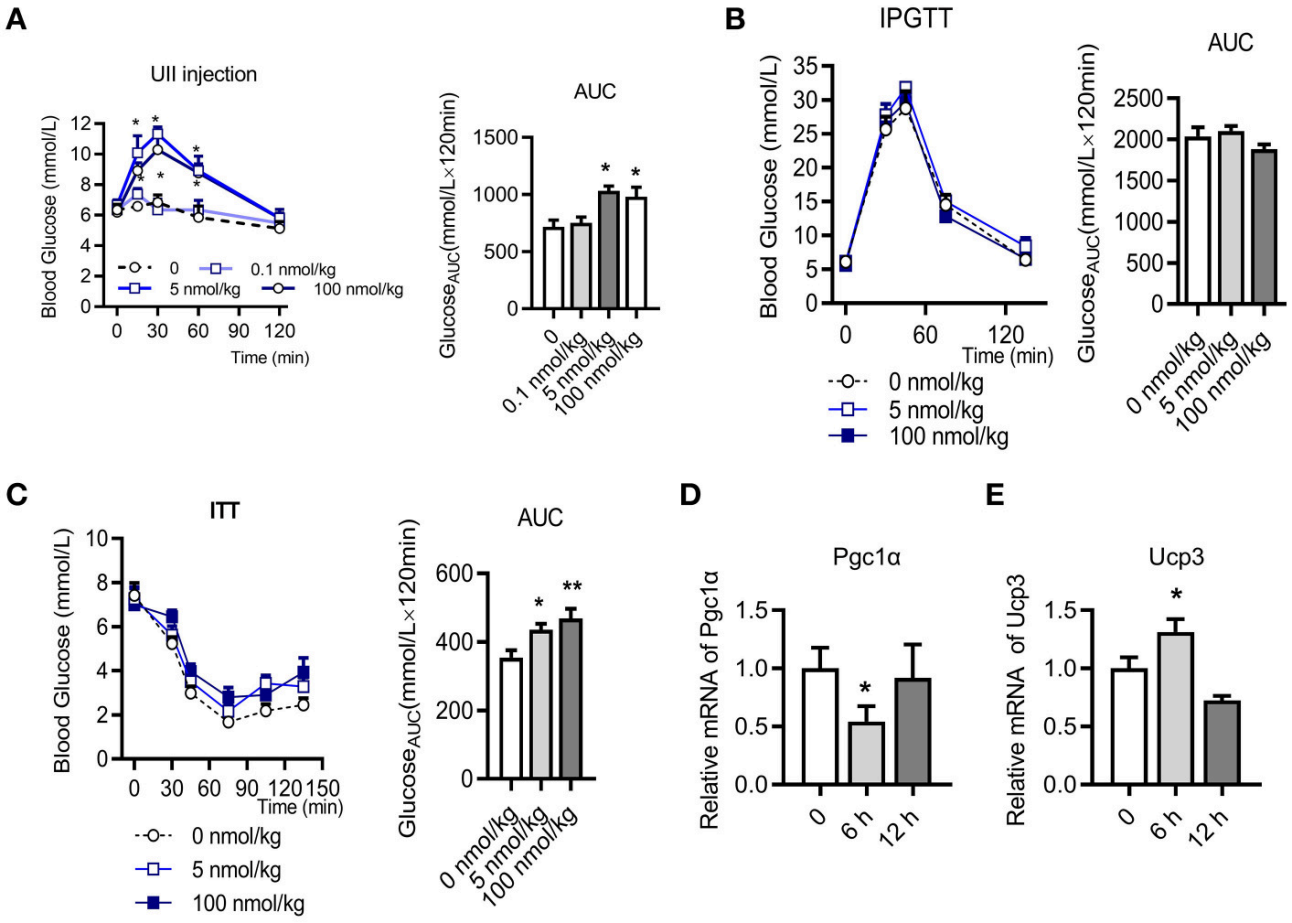
Urotensin-II single administration increased blood glucose and caused insulin resistance in chow diet-fed C57BL/6J mice. **(A)** Effect of urotensin-II (0.1, 5, and 100 nmol/kg) after IP administration on blood glucose. Left: blood-glucose curve. Right: AUC of blood glucose. **(B)** Effect of urotensin-II (5 and 100 nmol/kg) after IP administration on IPGTT. Left: blood-glucose curve. Right: AUC of blood glucose. **(C)** Effect of urotensin-II (5 and 100 nmol/kg) after IP administration on ITT. Left: blood-glucose curve. Right: AUC of blood glucose. **(D)** Effect of urotensin-II (100 nmol/kg) after single IP administration on the *Pgc1*α gene mRNA expression level in skeletal muscle after administration for 6 h. **(E)** Effect of urotensin-II (100 nmol/kg) after single IP administration on *Ucp3* gene mRNA expression level in skeletal muscle after administration for 6 h. Data are represented as the means ± SEM, *n* = 7; biological replicates. **(A–C)** Statistical analysis tested by two-way ANOVA with Dunnett's multiple comparisons test analysis; **(D,E)** Statistical analysis tested by one-way ANOVA with Dunnett's multiple comparisons test analysis. ^*^P < 0.05, ^**^P < 0.01 for compared with control.

To evaluate the effects of U-II on the blood-glucose levels, we first investigated U-II (0.1–100 nmol/kg) single administration in chow diet-fed mice. Our results showed that U-II at concentrations of 5 and 100 nmol/kg increased blood glucose within 60 min (Figure 1A). After U-II administration for 15 min, the IPGTT or ITT was performed (*N* = 7 in each group). U-II did not significantly affect glucose tolerance (Figure 1B). U-II treatments (5 and 100 nmol/kg) led to significant insulin resistance (Figure 1C). Single administration of 100 nmol/kg U-II reduced α subunit of peroxisome proliferators activated receptor γ coactivator-1(*Pgc1*α) expression (Figure 1D) but induced uncoupling protein 3 (*Ucp3*) expression in skeletal muscle (Figure 1E).

### Chronic U-II Administration Ameliorated Glucose and Insulin Tolerance in C57BL/6J Mice

**Figure 2:**
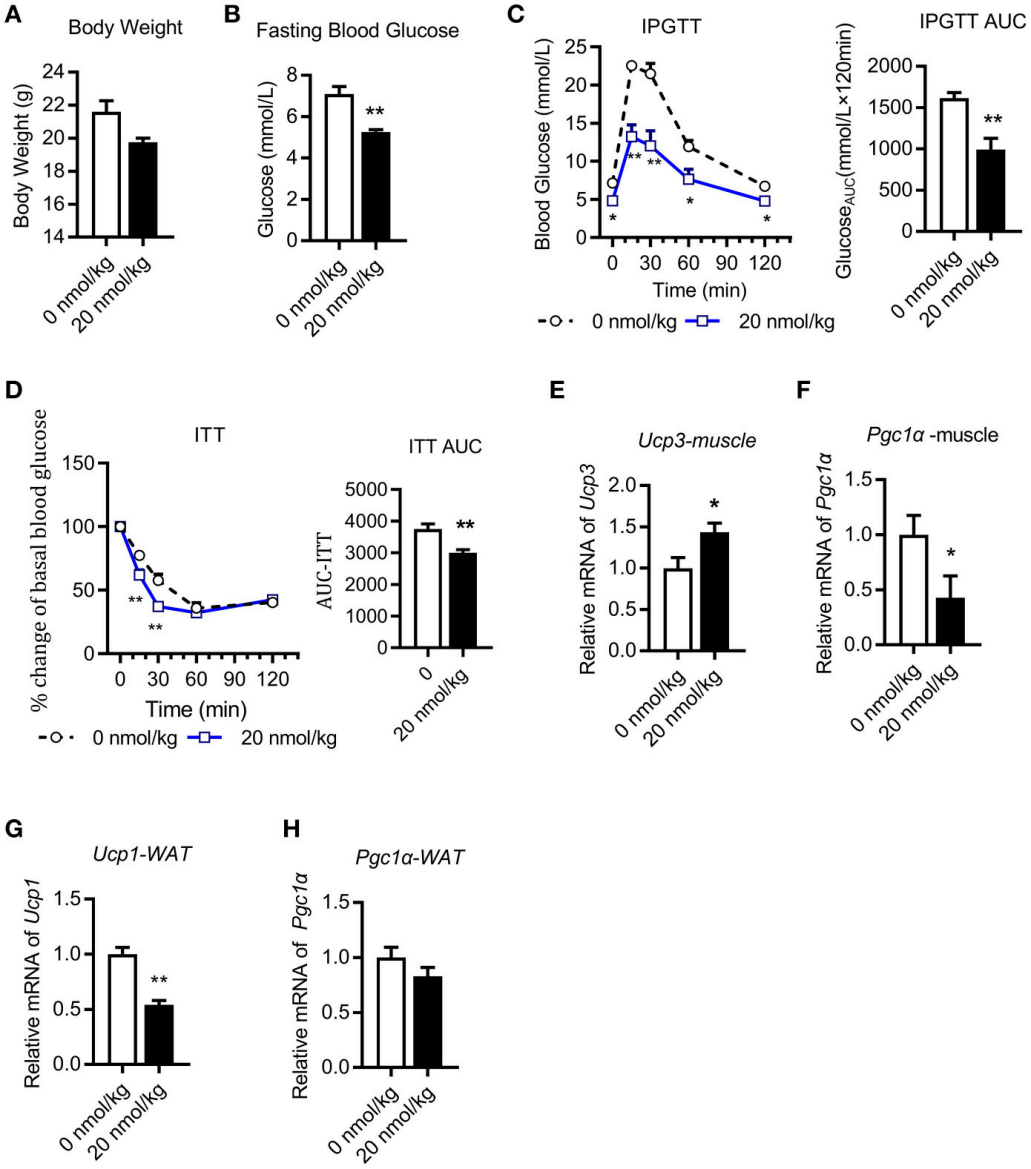
Chronic urotensin-II administration ameliorated glucose and insulin tolerance in C57BL/6J mice. Urotensin-II administration once a day by IP for seven days in chow diet mice. **(A)** Body weight after seven-day urotensin-II treatment. **(B)** Fasting blood glucose after seven-day urotensin-II treatment. **(C)** Effect of urotensin-II (20 nmol/kg) after IP administration on IPGTT. Left: blood-glucose curve. Right: AUC of blood glucose. **(D)** Effect of urotensin-II (20 nmol/kg) after IP administration on ITT. Left: blood-glucose curve. Right: AUC of blood glucose. **(E–H)** Effect of chronic of urotensin-II (20 nmol/kg) IP administration on gene mRNA expression in tissues. **(E)** *Ucp3* gene in skeletal muscle. **(F)** *Pgc1*α gene in skeletal muscle. **(G)** *Ucp1* gene in white fat tissue. **(H)** *Pgc1*α gene in white fat tissue. Data are represented as the means ± SEM, *n* = 5; biological replicates. **(C,D)** Statistical analysis tested by two-way ANOVA with Dunnett's multiple comparisons test analysis; **(A–H)** Statistical analysis was performed by unpaired 2-tailed Student's *t*-tests. ^*^P < 0.05, ^**^P < 0.01 for compared with control.

We next performed chronic U-II (20 nmol/kg) administration in the chow diet-fed C57BL/6J mice. After 7 days of administrations, the body weights of mice in the U-II treatment group were significantly reduced compared with those in the control group (Figure 2A). Fasting blood glucose decreased significantly in the U-II treatment group (Figure 2B). Glucose tolerance increased in the U-II treatment group (Figure 2C), while insulin sensitivity improved (Figure 2D). *Ucp3* expression was significantly induced in skeletal muscle (Figure 2E). Chronic U-II administration also reduced *Pgc1*α gene expression in skeletal muscle (Figure 2F). However, the *Ucp1* level in white fat tissue was significantly reduced (Figure 2G) while *Pgc1*α didn't change (Figure 2H).

### Chronic U-II Administration Ameliorated Glucose Tolerance in HFD-Fed C57BL/6J Mice

**Figure 3:**
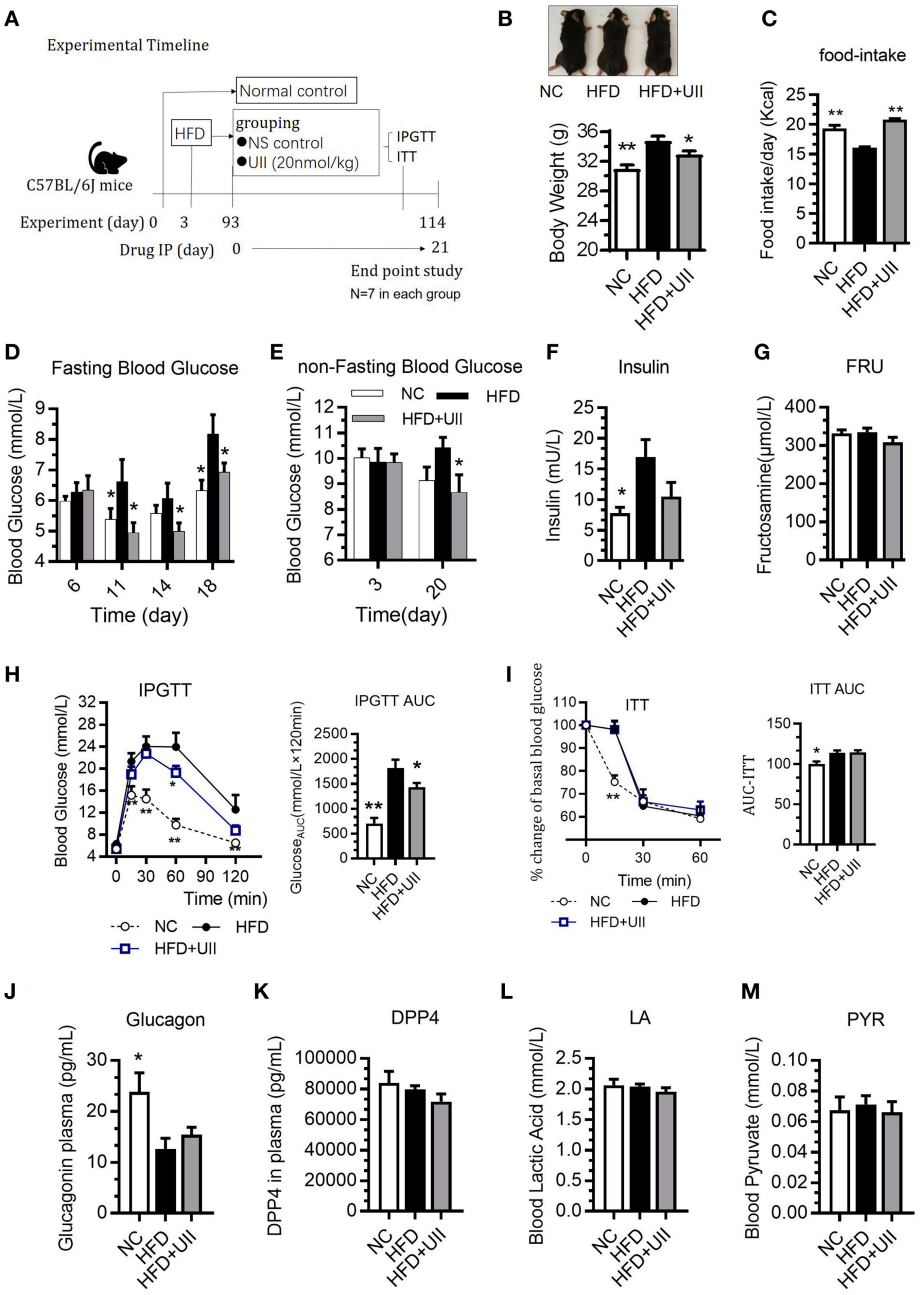
Chronic urotensin-II administration ameliorated glucose tolerance in HFD C57BL/6J mice. **(A)** Experimental timeline: urotensin-II administration once a day for 21 days by IP. **(B)** Body weight after 21 days of U-II treatments. **(C)** Food intake. **(D)** Fasting blood glucose. **(E)** Non-fasting blood glucose. **(F)** Insulin level. **(G)** Blood fructosamine level. **(H)** IPGTT. Left: blood-glucose curve. Right: AUC of blood glucose. **(I)** ITT. Left: blood-glucose curve. Right: AUC of blood glucose. **(J)** Blood glucagon. **(K)** Blood DPP4. **(L)** Blood lactic acid. **(M)** Blood pyruvate. Data are represented as the means ± SEM, *n* = 7; biological replicates. **(H,I)** Statistical analysis tested by two-way ANOVA with Dunnett's multiple comparisons test analysis; **(B–M)** Statistical analysis tested by one-way ANOVA with Dunnett's multiple comparisons test analysis. ^*^P < 0.05, ^**^P < 0.01 for compared with HFD control.

HFD-fed mice were administered U-II (20 nmol/kg, IP) for 21 days (Figure 3A). Compared with that of the chow-diet control, the weight of the mice increased in the HFD group. U-II administration decreased body weight (Figure 3B) but increased food intake (Figure 3C) in HFD mice. Fasting blood-glucose levels increased in HFD-fed mice, and U-II administration reduced fasting blood-glucose levels (Figure 3D) and non-fasting blood-glucose levels (Figure 3E). Nonetheless, U-II did not significantly affect insulin levels (Figure 3F) or fructosamine levels (Figure 3G). U-II administration increased glucose tolerance (Figure 3H) but without insulin sensitivity change (Figure 3I) in HFD-fed mice. U-II had no significant effect on blood glucagon (Figure 3J), dipeptidyl peptidase-4 (DPP4) (Figure 3K), lactic acid (Figure 3L), or pyruvate (Figure 3M).

### Chronic U-II Administration Reduced Fat Tissue Weight but Had No Effect on Blood Lipids

**Figure 4:**
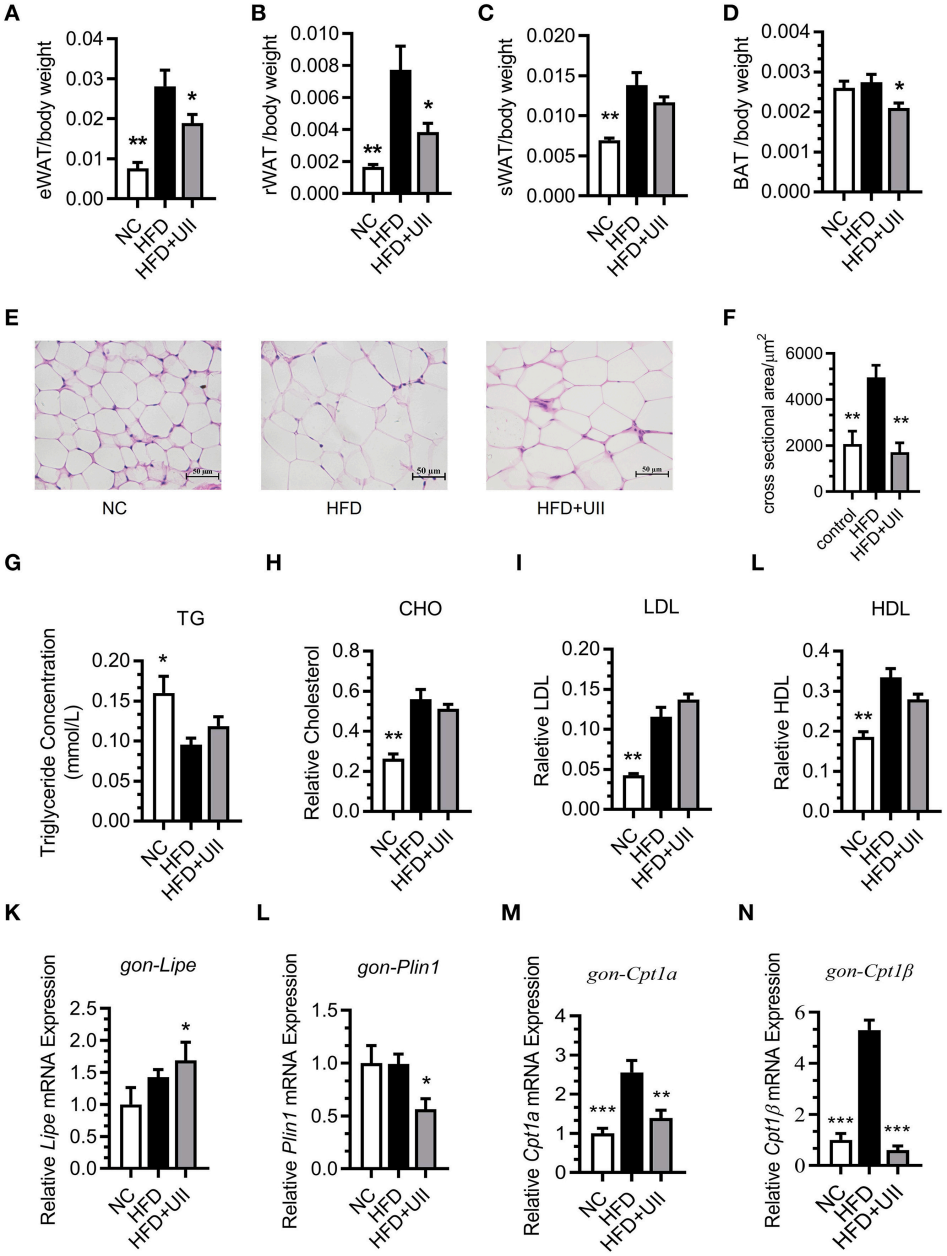
Chronic urotensin-II administration reduced fat tissue weight but had no effect on blood lipid levels. **(A–D)** Fat mass index of epididymal adipose (eWAT), retroperitoneal adipose (rWAT), subcutaneous white adipose tissue (sWAT), and brown adipose tissue (BAT) of HFD mice treated with uroteinsin-II for 21 days. **(E)** Representative images (five total images per group) of H&E staining of eWAT. **(F)** Adipose cell size of eWAT (five total images per group). **(G)** Blood triglyceride levels. **(H)** Blood CHO levels. **(I)** Blood LDL levels. **(J)** Blood HDL levels. **(K–N)** Relative mRNA expression levels of genes involved in lipolysis and fatty acid oxidation in eWAT. **(K)** *Lipe* mRNA. **(L)** *Plin1* mRNA. **(M)** *Cpt1*α mRNA. **(N)** *Cpt1*β mRNA. Scale bars, 50 μm. Data are represented as the means ± SEM, *n* = 7; biological replicates. Statistical analysis tested by one-way ANOVA with Dunnett's multiple comparisons test analysis. ^*^P < 0.05, ^**^P < 0.01, ^***^P < 0.001 for compared with HFD control.

Our results showed that the weights of several types of adipose tissue were significantly reduced in the U-II treatment group after 3 weeks of U-II treatments (Figures 4A–D), including epididymal adipose (eWAT) (Figure 4A), retroperitoneal adipose (rWAT) (Figure 4B), subcutaneous white adipose tissue (sWAT) (Figure 4C), and brown adipose tissue (BAT) (Figure 4D). The adipose cells were smaller under U-II treatment compared with HFD control (Figures 4E-G). Treated with 20 nmol/kg U-II for 21 days had no significant effect on blood lipid levels (Figures 4G–J). U-II induced the expression of the key lipolytic gene *Lipe* (Figure 4K) and reduced the expression of the lipid storage gene *Perilipin1* (Figure 4L) in eWAT. U-II also reduced expression of the fatty acid oxidation gene *Cpt1* in eWAT compared with that in HFD-fed mice (Figures 4M,N).

### U-II Increased Mitochondrial Ucp3 Expression in Gastrocnemius Muscle

**Figure 5:**
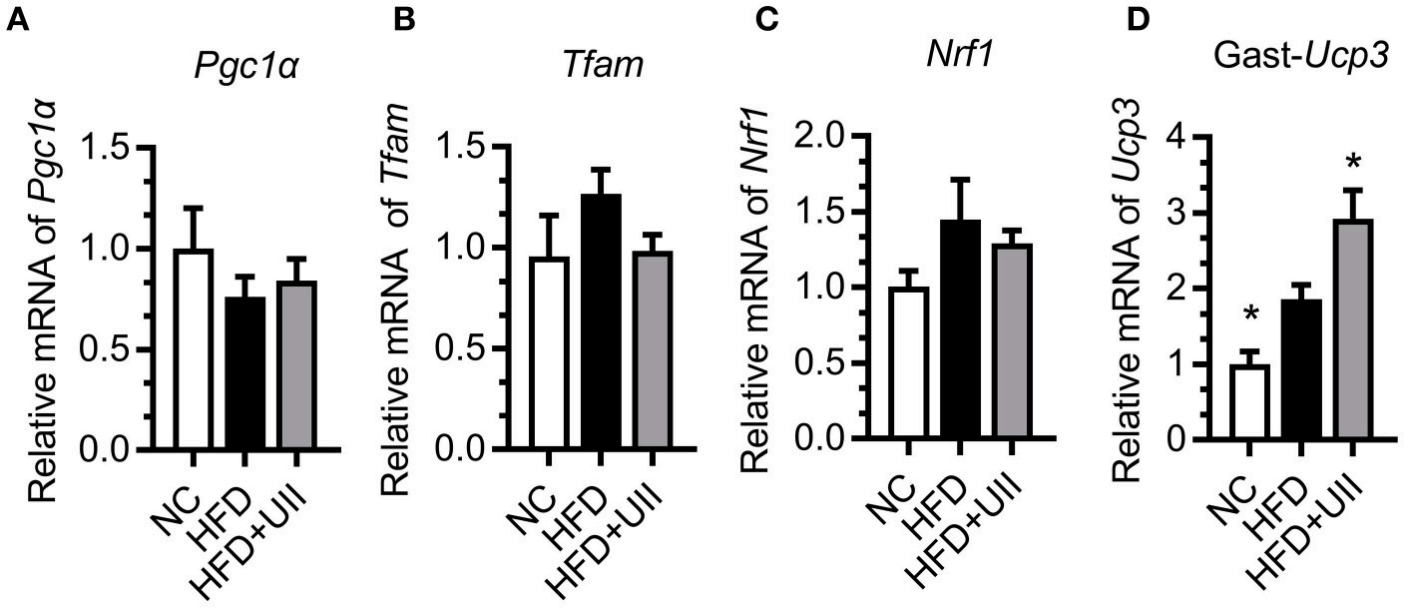
Urotensin-II increased mitochondrial *Ucp3* expression in gastrocnemius muscle of HFD-fed mice. **(A–C)** Mitochondrial biosynthesis gene mRNA expression. **(A)** *Pgc1*α, **(B)** *Tfam*, **(C)** *Nrf1*, **(D)** *Ucp3*. Data are represented as the means ± SEM, *n* = 7; biological replicates. Statistical analysis tested by one-way ANOVA with Dunnett's multiple comparisons test analysis. ^*^P < 0.05 for compared with HFD control.

Skeletal muscle is the primary tissue for glucose and fatty acid use and energy production (24). Our results showed that U-II did not affect the mitochondrial biosynthesis in the gastrocnemius muscle of HFD-fed mice (Figures 5A–C). Uncoupling proteins are mitochondrial inner membrane proteins that are essential for thermogenesis and maintaining body temperature. In this study, U-II increased *Ucp3* expression in HFD-fed mice (Figure 5D).

### U-II Increased Aerobic Respiration in Skeletal Muscle Cells

**Figure 6:**
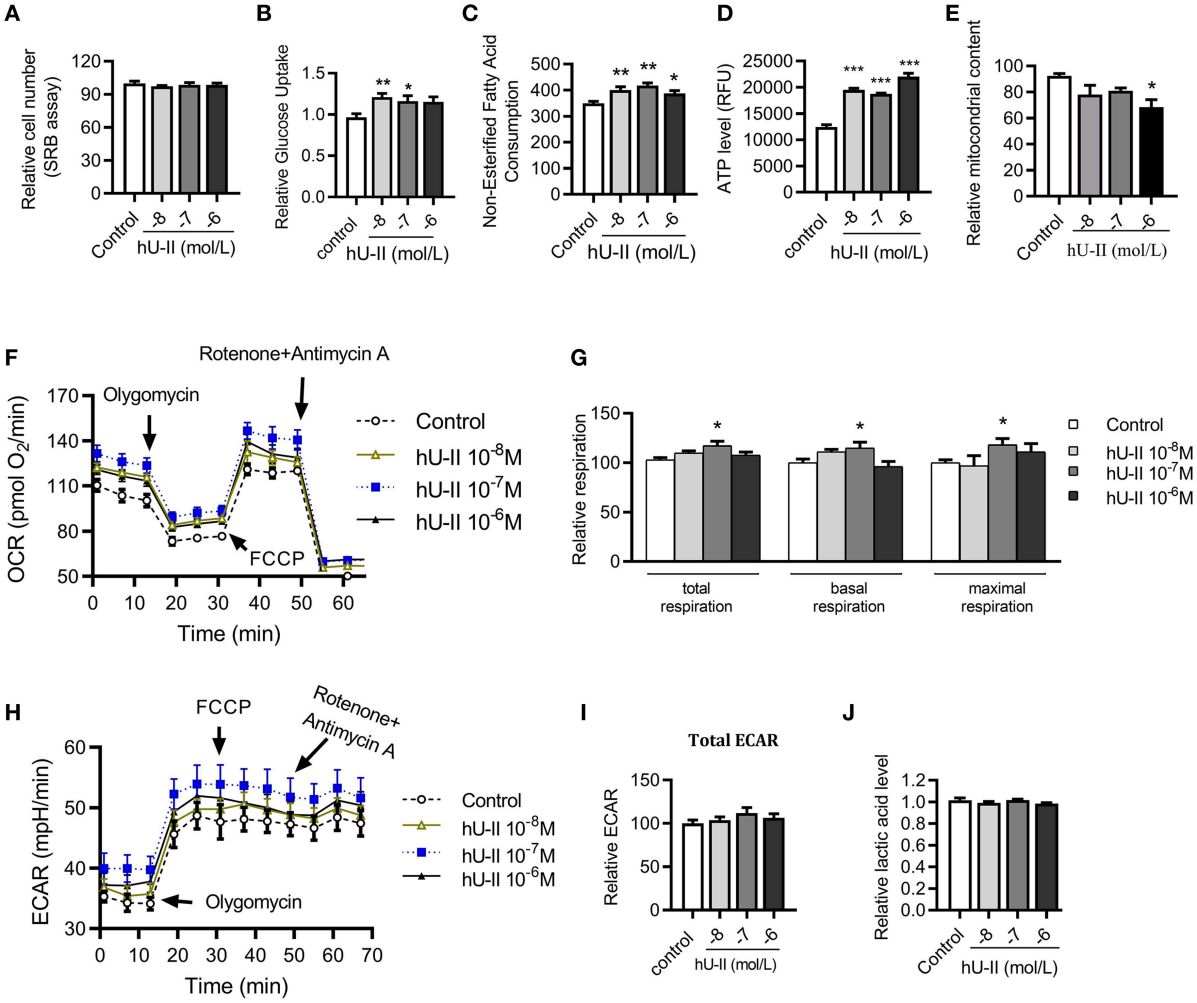
Urotensin-II increased glucose and FFA consumption and increased aerobic respiration *in vitro*. C2C12 myoblasts were treated with various concentrations of urotensin-II (10^−8^-10^−6^ M) for 24 h. **(A)** Cell number measured by sulforhodamine B assay. **(B)** Glucose uptake. **(C)** Fatty acid consumption. **(D)** ATP production. **(E)** Mitochondria copy number measured by MitoTracker probe. **(F)** Oxygen consumption rate (OCR) curve. **(G)** Total oxygen consumption rate, basal oxygen respiration rate, and maximal oxygen respiration rate. **(H)** Extracellular acidification rate (ECAR) curve. **(I)** Total ECAR. **(J)** Relative lactic acid levels in the supernatant. The data shown are representative of three experiments. Data are represented as the means ± SEM, *n* = 6; biological replicates. Statistical analysis tested by one-way ANOVA with Dunnett's multiple comparisons test analysis. ^*^P < 0.05, ^**^P < 0.01, ^***^P < 0.001 for compared with control.

To further clarify the effect and mechanism of U-II on skeletal muscle, C2C12 muscle cells were employed to evaluate the effect of U-II on mitochondrial function. Treated with various concentrations of U-II (10^−8^-10^−6^ mol/L) for 24 h, there was no significant change on the cell number (Figure 6A). U-II (10^−8^-10^−7^ mol/L) increased glucose uptake (Figure 6B). U-II (10^−8^-10^−6^ mol/L) also increased FFA consumption (Figure 6C). U-II (10^−8^-10^−6^ mol/L) significantly increased cellular ATP production (Figure 6D). However, U-II (10^−6^ mol/L) reduced the cellular mitochondrial copy numbers (Figure 6E). After incubation for 24 h, U-II (10^−7^ mol/L) significantly increased the total, basal and maximal oxygen consumption rates (Figures 6F,G), whereas U-II had no significant effect on ECAR (Figures 6H,I) and did not influence lactic acid production (Figure 6J).

## Discussion

**Figure 7:**
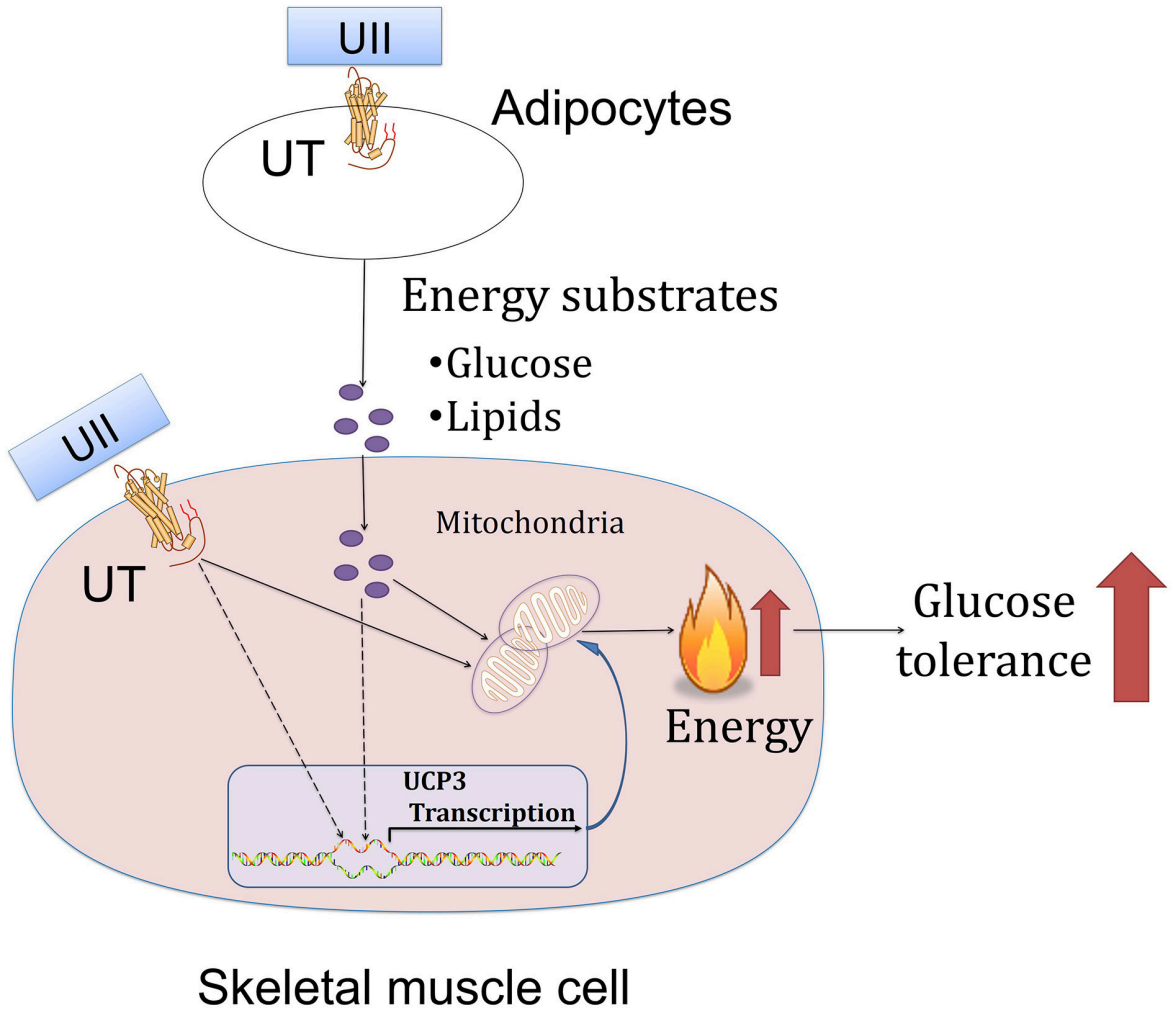
A schematic diagram illustrating the proposed mechanism of urotensin-II.

In this study, we showed that chronic U-II administration could improve glucose tolerance in both chow-diet- and HFD-induced obese mice. This robust effect in U-II-treated mice was manifested by a reduced adipose tissue weight index, increased *Ucp3* gene expression in skeletal muscle and decreased blood-glucose level (Figure 7). In addition, our results also showed that U-II could also induce an initial (60 min) transient hyperglycemia and insulin resistance.

In this report, we first demonstrated that chronic U-II administration could significantly ameliorate glucose tolerance in mice. This report is consistent with a previous report that U-II tended to induce hypoglycemia in coho salmon fish (16). In contrast, this result is different from previous reports that blocking the UT receptor pathway ameliorates metabolic syndrome in mice (12, 15). U-II administration with a dosage of 500 pmol/kg body weight per hour for a total of 4 weeks had no significant effect on glucose levels (18).

Consistent with a previous study revealing that U-II can increase food consumption under intracerebroventricular administration (6, 25), we observed that food intake increased under U-II administration. Our study also showed that body weight dropped. An increase in food intake with a decrease in body weight suggests an increase in energy expenditure in mice.

The important organs for the regulation of insulin sensitivity and energy expenditure are adipose tissue, liver and skeletal muscle (26). It is conceivable that U-II could have effects on these tissues. Indeed, a previous study suggests that the UTS2 gene may regulate fat accumulation in humans (27). Our study further confirmed the speculation that U-II significantly decreased various types of adipose tissue weights. This is in contrast with a previous report that the *Uts2* gene deletion in mice reduced visceral fat (11).

The initial event triggers adipose tissue size shrinkage involving energy expenditure and lipid uptake and distribution. Uncoupling proteins are mitochondrial anion carrier proteins. UCP1 plays the main thermogenic role in adipose tissue (28). In contrast to a previous study in which U-II increased BAT *Ucp 1* mRNA expression (29), we found that intraperitoneal injection administration of U-II (20 nmol/kg) decreased adipose tissue *Ucp1* expression, which suggests that the reduced adipose weight was not due to adipose thermogenesis since adipocyte numbers are roughly constant in lean and obese adults (30). It is theoretically possible that lipid mobilization may play an important role in this phenomenon. Indeed, in this study, we found U-II led to lipid mobilization through promoting adipose tissue lipolysis. We found that U-II modulated key lipolysis genes—induced *Lipe* and reduced *Plin 1* expression in adipose tissue. *Lipe* is closely related to lipolysis and *Plin 1* inhibits lipolysis (31, 32). We also found that U-II increased liver and spleen weight (data not shown), which suggests that ectopic fat existed. This finding is consistent with previous findings that U-II directly enhanced lipid mobilization from salmon liver slices and stimulated the activity of triacylglycerol lipase and the release of FFAs in the liver in salmon fish (16, 33). Our results contribute to the novel discovery that U-II increases lipid mobilization in mice without blood lipid changes. However, the blood lipid profiles unchanged. We thus speculate that the excess lipids from lipid mobilization were metabolized within the body. Skeletal muscle is an important organ involved in lipid metabolism (26, 34, 35). The majority of fatty acids delivered to skeletal muscle are derived from adipose tissue (36). Our study suggests that UII promotes adipocytes releasing lipids, which then consumed by skeletal muscle, and thus maintain blood lipid levels.

The mitochondrion is a key place in cells for glucose metabolism. However, until now, there has been no report on the effects of U-II on the mitochondrion. Our study found that U-II did not affect mitochondrial biosynthesis gene expression in HFD-fed mice, which means that U-II did not significantly affect the mitochondria number. This effect was further confirmed by the *in vitro* study in myotubes. Glucose and FFA are the main sources of skeletal muscle energy production. We found that U-II (10^−8^-10^−7^ M) increased glucose and FFA consumption with augmented oxygen consumption. Our study showed that U-II increased *Ucp3* gene expression in skeletal muscle. *Ucp3* is an important mitochondrial anion carrier protein and shows highly selective expression in skeletal muscle; it mitigates reactive oxygen species (ROS) production and is involved in fatty acid oxidation (37). The effects of U-II on *Pgc1*α, *Ucp3* expression in skeletal muscle were consistent under acute and chronic treatments, and they were not influenced by the difference of blood-glucose level.

We observed that single administration of U-II could induce insulin resistance immediately. This result is consistent with previous reports that U-II induced insulin resistance (38) and reduced glucose-evoked insulin release (6, 14). We also found that chronic U-II administration didn't improve HFD-induced insulin resistance, which may because U-II didn't change HFD-induced dyslipidaemia. In this study, U-II induced a rapid blood-glucose elevation. This result agrees with a previous study in which U-II induced hyperglycemia following intracerebroventricular injection (39). We speculate this effect may be achieved by promoting hepatic glycogen decomposition which is needed further investigation. Results from our study suggest that U-II has a two-edge effect and that U-II could induce an initial transient hyperglycemia. However, chronic U-II administration (>1 week) tended to ameliorate glucose tolerance. Because of these effects, U-II may have limited potential as a hypoglycemia drug. However, it can reduce the weight of adipose tissue and improve glucose tolerance by long-term application, which makes its application in other metabolic disorders such as obesity and pre-diabetic state more promising. Whereas, more investigations are still required due to some paradoxical effects. The understanding of U-II on glucose and lipid metabolism, along with its direct effects on mitochondria in myocytes, not only facilitates a better understanding of the function of the urotensinergic system but also promotes drug development.

In conclusion, our study reports for the first time that U-II, as an endogenous ligand of the urotensinergic system, can ameliorate glucose in HFD-induced obesity in mice. This effect may be closely related to reduced-fat tissue and increased glucose and fatty acid oxidation in skeletal muscle.

## Data Availability

The datasets generated for this study are available on request to the corresponding author.

## Ethics Statement

All animal procedures were approved by the animal care and use committee of the Institute of Materia Medica, Chinese Academy of Medical Sciences, and were carried out strictly in accordance with research guidelines for the care and use of laboratory animals.

## Author Contributions

XC, LY, WJ, NW, CX, BH, NL, GQ, and XY performed the experiments. XC, LY, WJ, and XY performed statistical analysis. XY, LZ, GQ, and GD contributed to the experimental design and manuscript preparation. XY and GD conceived the project and gave the final confirmation of the manuscript. All authors have given approval to the final version of the manuscript.

### Conflict of Interest Statement

The authors declare that the research was conducted in the absence of any commercial or financial relationships that could be construed as a potential conflict of interest.
